# Semi-invasive aspergillosis in an immunocompetent patient with Swyer-James-MacLeod Syndrome: a case report

**DOI:** 10.1186/1752-1947-4-153

**Published:** 2010-05-26

**Authors:** Sara MST Salgado, Carla A Costa, António A Bugalho, Júlio ANMQ Semedo, José C Ribeiro, Luís M Carreiro

**Affiliations:** 1 Pulido Valente Hospital, Alameda das Linhas de Torres, Lisbon, Portugal

## Abstract

**Introduction:**

Invasive and semi-invasive pulmonary aspergillosis usually occurs in immunocompromised patients. It has been described occasionally in patients with normal immunity and previous lung disease such as chronic obstructive pulmonary disease.

Swyer-James-MacLeod Syndrome is a rare condition characterized by hyperlucency of one lung, lobe or part of a lobe due to decreased vascularity and air trapping.

**Case presentation:**

We report a case of semi-invasive pulmonary aspergillosis in a 38-year-old Portuguese, Caucasian man who is immunocompetent, with a pre-existing Swyer-James-McLeod Syndrome, a structural lung disease.

**Conclusions:**

To the best of our knowledge, this is the first reported case in the literature on the relationship between these two diseases. Although rare, aspergillosis can occur in immunocompetent adults with a pre-existing lung disease other than chronic obstructive pulmonary disorder.

## Introduction

Invasive and/or semi-invasive aspergillosis infection is extremely rare in patients with normal immunity. It has been described in the presence of pulmonary disease, such as chronic obstructive pulmonary disorder (COPD), but it can also occur in patients without pre-existent disease, usually following massive inoculums of Aspergillus. Although rare it can be fatal [1-6].

## Case presentation

A 38-year-old Portuguese, Caucasian man working in the viticulture and forestry industry was referred to a pulmonary clinic following complaints of progressive right side pleuritic chest pain, non-productive cough, low-grade fever, and general fatigue.

He was a former smoker (10 packs a year) and had asymptomatic Swyer-James-MacLeod Syndrome (SJMS) that was diagnosed at the age of 28 after a routine chest X-ray. He also had arterial hypertension that was controlled with atenolol and amiloride plus hydrochlorothiazide. He also reported frequent exposure to organic dust during work.

A physical examination of our patient revealed normal body temperature, pulse rate, respiratory rate, blood pressure and oxygen saturation. His chest examination revealed crackles in his lower right hemithorax. The rest of his physical exam was unremarkable.

Blood sample analysis showed that he had no abnormalities except for an elevated erythrocyte sedimentation rate (ESR) (72 mm/h, normal: <20 mm/h) and C-reactive protein (CRP) (25.95 mg/dL, normal: <1 mg/dL). His lung function tests and arterial blood gas levels were normal.

**Figure 1:**
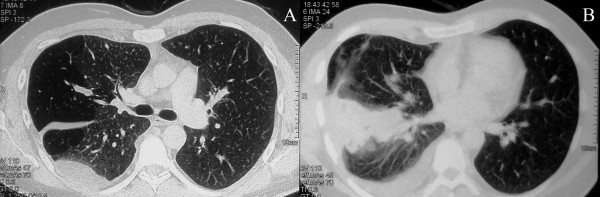
**Chest computed tomography scan**. **(A) **Areas of air-trapping with hyperlucency mainly in the right upper lobe (compatible with Swyer-James-MacLeod Syndrome). **(B) **A mass with pleural contact and a small pleural effusion in the right lung (semi-invasive aspergillosis).

A plain chest X-ray disclosed a large infiltrate in the right inferior lung field of our patient and a chest computed tomography (CT) scan confirmed the presence of a consolidation and/or a mass of 35 × 64 × 37 mm in diameter located in the upper segment of the right lower lobe and posterior segment of his right upper lobe. The mass had direct contact with the contiguous pleura and was associated with a small pleural effusion, and there was also no mediastinal lymphadenopathy (Figure 1).

We initiated a therapy of levofloxacin 500 mg/day for one week. However, our patient showed no clinical response to this treatment.

Bronchoscopy was subsequently performed on our patient. His bronchoalveolar lavage (BAL) fluid revealed negative cytological, bacteriological, mycobacteriological and mycological exams. Bronchial brushing and transbronchial biopsies were also obtained and were negative for neoplastic cells.

A transthoracic CT-guided fine needle aspiration of the lesion was negative for neoplastic cells, but revealed fungal elements.

Finally, surgical lung biopsies were performed, which showed evidence of tissue invasion by fungal organisms and Sabouraud glucose agar cultures isolated multiple *Aspergillus glaucus *colonies. Serological tests did not reveal elevated titres of Aspergillus antibody or antigen (Platelia™ Aspergillus EIA, Bio-Rad).

Human immunodeficiency virus 1 and 2 tests, as well as differential cell counts for lymphocyte subpopulations, were performed on our patient and excluded underlying immunosuppression.

**Figure 2:**
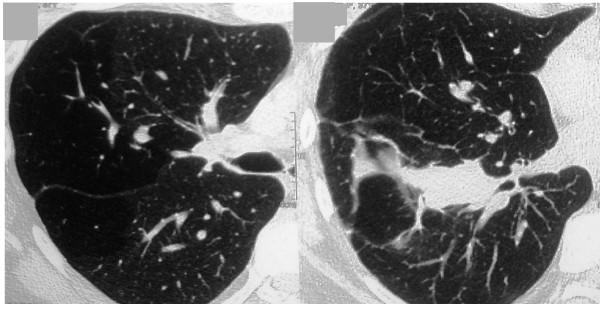
**Imaging after treatment**. Computed tomography scan demonstrates a decrease in the lesion size.

Our final diagnosis was semi-invasive pulmonary aspergillosis and we started him on itraconazole (400 mg orally per day) for one year. During follow-up examination he had remained asymptomatic and repeated chest CT revealed partial regression of the mass volume and resolution of the pleural effusion (Figure 2). His recent BAL fluid examination presented positive galactomannan and negative mycological exam. Blood analysis showed normalization of his ESR and CRP level.

He maintains treatment with itraconazol and is under deliberation for surgical resection of the residual lesion.

## Discussion

SJMS is a rare condition and was first described in 1953. It is considered to be a post-infection form of bronchiolitis obliterans that develops after pulmonary infections in childhood. Its clinical manifestations may vary from asymptomatic forms, in which the diagnosis follows a radiological finding, to recurrent respiratory infections with productive cough, wheezing and occasional hemoptysis. Hyperlucency of the affected area (lung, lobe or part of a lobe), diminished size of pulmonary vessels, and air trapping are the usual findings in chest CT [7,8]. To some extent, SJMS may have common features with COPD, such as the existence of emphysema bullae, airflow obstruction, air trapping, and a predisposition to respiratory infections.

Once Aspergillus spores are inhaled they can cause lung infections ranging from saprophytic to invasive forms. Its clinical presentation depends on the immune status of the host and underlying lung diseases. In the case we describe here, our patient was immunocompetent but presented a pre-existing structural lung disease, SJMS, and a history of probable professional inhalation of fungal spores. The clinical picture was an indolent form with focal disease (semi-invasive).

The diagnosis of Aspergillus infection is not always easy as it requires detection of Aspergillus in cultures and/or demonstration of tissue invasion by the fungus in the histological exam. In most cases, the diagnosis is made by tissue isolation through invasive methods, as in this case. Serological tests are adjuncts to support or exclude the diagnosis in the appropriate clinical context but they do not make a definitive diagnosis. In some cases the serum antigen level may be below the threshold of detection and antibody titration has a limited value.

Treatment is most often prolonged or combined [9,10]. The choice of the anti-fungal was based on the normal immune status and non-severity of the infection, which allows for oral treatment. Itraconazole is a reasonable drug for patients who are immunocompetent, with non-life-threatening forms of aspergillosis. It is also less expensive than voriconazole (the first-line agent), more comfortable and easily accessible for patients as it is available in non-hospital pharmacies. Also, our patient had no concomitant medication that would predict drug interaction problems. Finally, there are no reports of itraconazole resistant *A. glaucus *as this type of resistance is only described for *A. fumigatus*.

Our patient showed clinical and radiological improvement with itraconazole treatment. Our patient's age, good functional status and the lesion's reduction with medical treatment suggests that he might be a good candidate for surgical resection, although another option could be to prolong his itraconazole medication until the complete radiological resolution of his lesions.

## Conclusions

This is an original case report of interest to pulmonary and infectious disease specialities.

To the best of our knowledge, this is the first instance in the literature that the fact that semi-invasive aspergillosis can occur in immunocompetent adults with pre-existing lung disease other than COPD is highlighted. It is known that SJMS predisposes patients to lung infections and has also some similarities with COPD that could point out to potential fungal infections.

In such patients, prevention of environmental exposure should be attempted. Patients should be advised to use mechanical filter masks if potential spore inhalation exists and should be clinically monitored. Infections should be diagnosed and treated as soon as possible. Aspergillosis should be considered when an aetiological agent is not identifiable in a patient who fails to respond to antimicrobial agents.

## Competing interests

The authors declare that they have no competing interests.

## Authors' contributions

LC is the chief of the Pulmonology unit of our hospital and performed the initial evaluation of our patient. SS and CC followed up our patient. AB, JS and JCR performed the invasive techniques that allowed the final diagnosis and specific treatment. SS and CC were the main writers of the article. AB reviewed the preliminary drafts of the manuscript and selected and improved the images presented in this report. JS and JCR made the final revision of the manuscript. LC performed the final editing of the manuscript. All authors read and approved the final manuscript.

## Consent

Written informed consent was obtained from our patient for publication of this case report and any accompanying images. A copy of the written consent is available for review by the Editor-in-Chief of this journal.
